# Long-term phenotypic selection of Akkaraman sheep to establish a sustainable dairy population under semi-arid conditions in Türkiye

**DOI:** 10.1007/s11250-026-05057-y

**Published:** 2026-05-12

**Authors:** Afşin Kocakaya, Büşra Yaranoğlu, Ömer Faruk Güngör, Mücahit Kahraman, Bora Özarslan, Evren Erdem, Necmettin Ünal, Ceyhan Özbeyaz, Halil Akçapınar

**Affiliations:** 1 Department of Animal Breeding and Husbandry, Faculty of Veterinary Medicine, Ankara University, Ankara, Türkiye; 2 Department of Animal Breeding and Husbandry, Faculty of Veterinary Medicine, Balıkesir University, Balıkesir, Türkiye; 3 Department of Veterinary, Vocational School, Bolu Abant İzzet Baysal University, Bolu, Türkiye; 4 Department of Animal Husbandry, Faculty of Veterinary Medicine, Harran University, Şanlıurfa, Türkiye; 5 Department of Animal Breeding and Husbandry, Faculty of Veterinary Medicine, Kırıkkale University, Kırıkkale, Türkiye

**Keywords:** Akkaraman, Dairy sheep, Within-breed selection, Milk yield, Semi-arid climate, Sustainability

## Abstract

This study aimed to assess the possibility of developing a dairy population of Akkaraman sheep following a long-term within-breed phenotypic selection program in semi-arid conditions in Türkiye. The study was conducted at Gözlü State Farm between 2012 and 2023 (12 years), covering approximately 3–4 generations, and included milk yield records from 6,822 purebred Akkaraman ewes. Milk yields were recorded on days 1, 45, 60, 75, 90, 105, and 120 of lactation. Quadratic spline interpolation based on these test-day means was used to reconstruct daily milk yield and to standardize lactation performance over a 120-day period; no formal genetic parameter estimation (e.g., BLUP, REML) was applied. Across the 12-year study period, the mean lactation milk yield reached 119.15 kg, with a mean daily milk yield of 0.99 kg, representing approximately a 2.5-fold increase compared with historical reports for the Akkaraman breed. The lactation curve exhibited a gradual peak followed by a sustained production phase, indicating a shift toward a more dairy-type lactation pattern. The results indicate that continuous within-breed phenotypic selection significantly enhances milk production in a fat-tailed, dual-purpose sheep breed without crossbreeding while preserving adaptation to semi-arid production conditions. The findings show that local sheep breeds can help create environmentally friendly and sustainable dairy production in areas with limited resources.

## Introduction

The global human population has increased rapidly over the past five decades, largely due to advances in healthcare and technology, and it is projected to continue growing at an accelerated rate. The Food and Agriculture Organization (FAO) says that by 2050, the world’s population will be 9.7 billion. This means that providing enough healthy food will be a major global problem (FAO 2012). In this context, milk and dairy products play a key role in ensuring food and nutritional security.

Sheep milk is a particularly valuable nutritional resource because it has high levels of protein, fat, energy, minerals, and vitamins, and its significance in human nutrition has been well documented (Balthazar et al. 2017; Mohapatra et al. 2019; Li et al. 2022; OECD/FAO 2023). Compared with human, horse, donkey, cow, goat, camel, llama, and yak milk, sheep milk contains higher concentrations of calcium, phosphorus, protein, fat, folic acid, and vitamins B and C (Claeys et al. 2014; Duman et al. 2024). In addition, sheep milk has been associated with beneficial effects on the circulatory system and cardiovascular health. As the world drinks more milk and dairy products, the demand for sheep milk has also been slowly rising (Duman et al. 2024).

However, the expansion of ruminant livestock populations contributes to global warming through increased greenhouse gas emissions, particularly methane. One of the most effective strategies to mitigate emissions from livestock systems is to improve animal productivity, thereby reducing methane emissions per unit of product. Higher-producing animals allocate a greater proportion of feed energy toward production, which results in a lower methane output per unit of milk, while also reducing the total number of animals required to meet production targets (Özbeyaz and Kocakaya 2011). Consequently, productivity-oriented breeding strategies play a key role in the development of climate-smart and sustainable livestock systems.

**Fig. 1:**
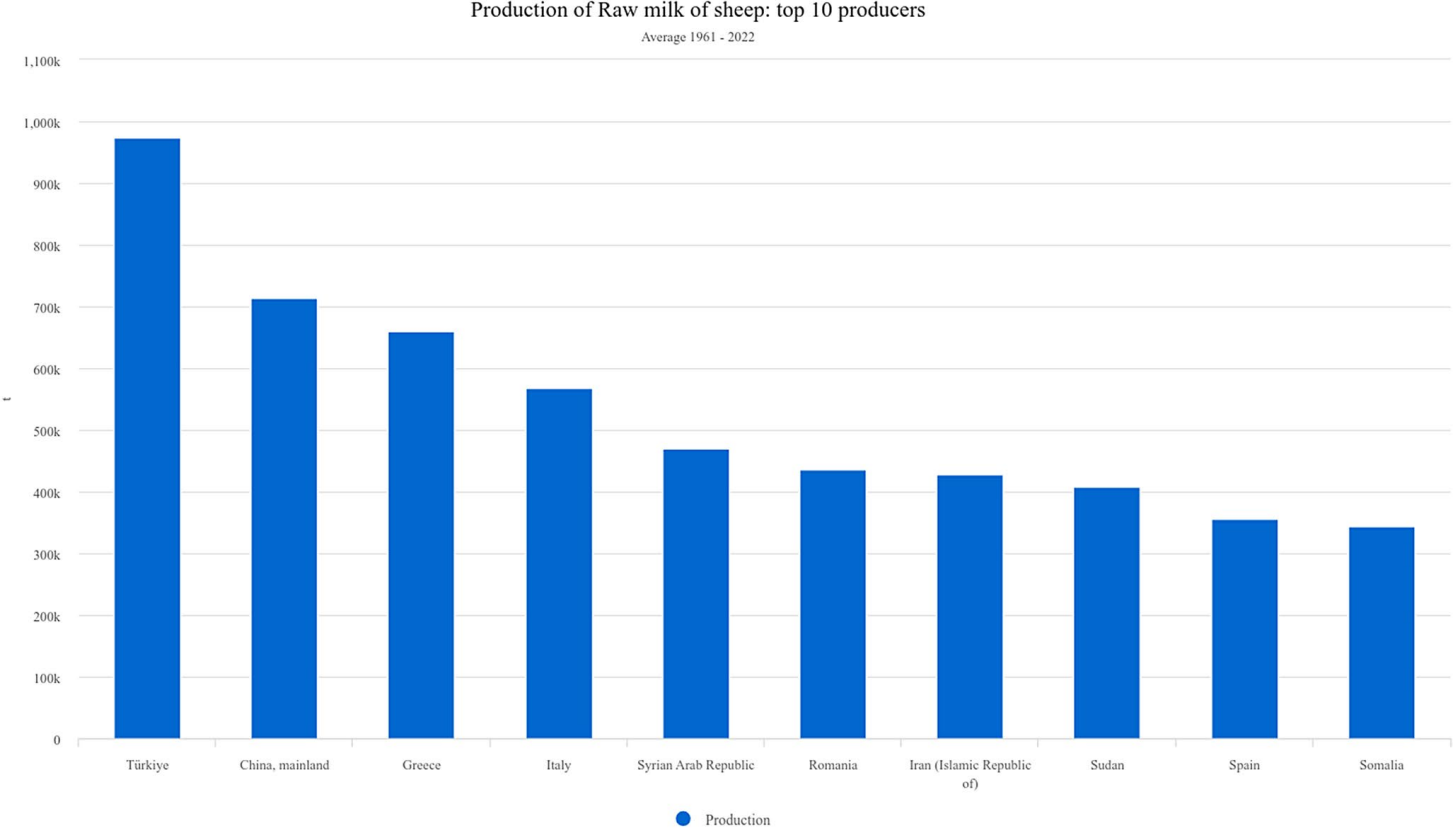
Top ten sheep milk–producing countries worldwide (1961–2022 average milk yield in 61 years), highlighting Türkiye’s historical leading position (FAOSTAT 2025)

**Fig. 2:**
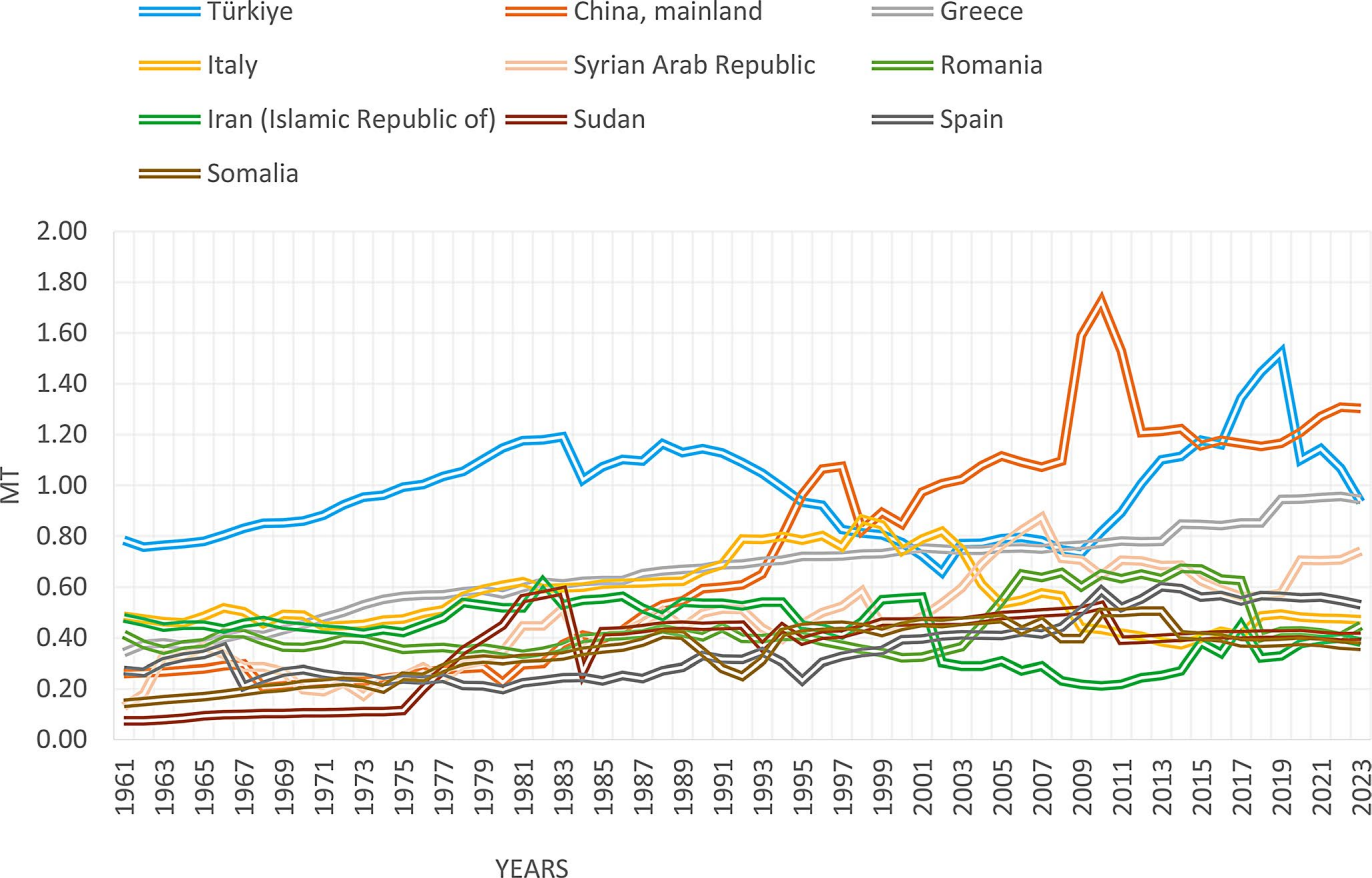
Annual sheep milk production of the top ten producing countries, illustrating Türkiye’s long-term trend and its shift relative to other major producers (FAOSTAT 2025). MT = Million Tonnes

Türkiye leads in sheep milk production when calculating the average of the 61 years between 1961 and 2022 (Fig. 1). While Türkiye was the leader in sheep milk production until the early 1990s, this situation changed in favor of China in the mid-1990s (Fig. 2) according to FAOSTAT data (FAOSTAT 2025). Current projections indicate that global milk production—dominated by cows (81%), followed by buffalo (15%) and small ruminants including sheep and goats (4%)—is expected to grow at an annual rate of approximately 1.5% over the next decade, reaching 1,039 million tonnes by 2032 (OECD/FAO 2023). Despite this growth, improvements in milk yield efficiency from locally adapted sheep breeds remain limited in many semi-arid regions.

These global trends provide the production context within which Türkiye’s historical and current position in sheep milk production can be evaluated (Figs. 1 and 2).

To address the dual challenges of food security and environmental sustainability, the “Improvement of the Akkaraman Breed through Pure and Crossbreeding” project was initiated in 2012 with the aim of enhancing milk production in the Akkaraman sheep, a fat-tailed breed well adapted to semi-arid conditions. Within this framework, the milk yield performance of Akkaraman sheep has been monitored and improved through long-term phenotypic selection. While crossbreeding with specialized dairy breeds can rapidly increase milk yield, it often compromises adaptation traits such as heat tolerance, disease resistance, and feed efficiency under extensive management (Akçapınar and Özbeyaz 2021; Güngör et al. 2022; Brandão et al. 2024). Therefore, within-breed selection offers a sustainable alternative to preserve genetic resilience while improving productivity. However, long-term selection carries the risk of narrowing genetic diversity, necessitating careful monitoring of inbreeding (Akçapınar and Özbeyaz 2021). Despite previous attempts to develop dairy lines in fat-tailed breeds, long-term empirical data on the feasibility of sustained phenotypic selection under semi-arid conditions remain limited.

We hypothesized that sustained within-breed phenotypic selection could significantly improve milk yield performance in the Akkaraman breed without compromising its adaptation to semi-arid production systems. Accordingly, the objective of this study was to evaluate the milk yield performance of the Akkaraman population developed at Gözlü State Farm over a 12-year selection period and to compare its performance with that reported for other local and international dairy sheep breeds.

## Materials and methods

### Flock description and selection strategy

**Fig. 3:**
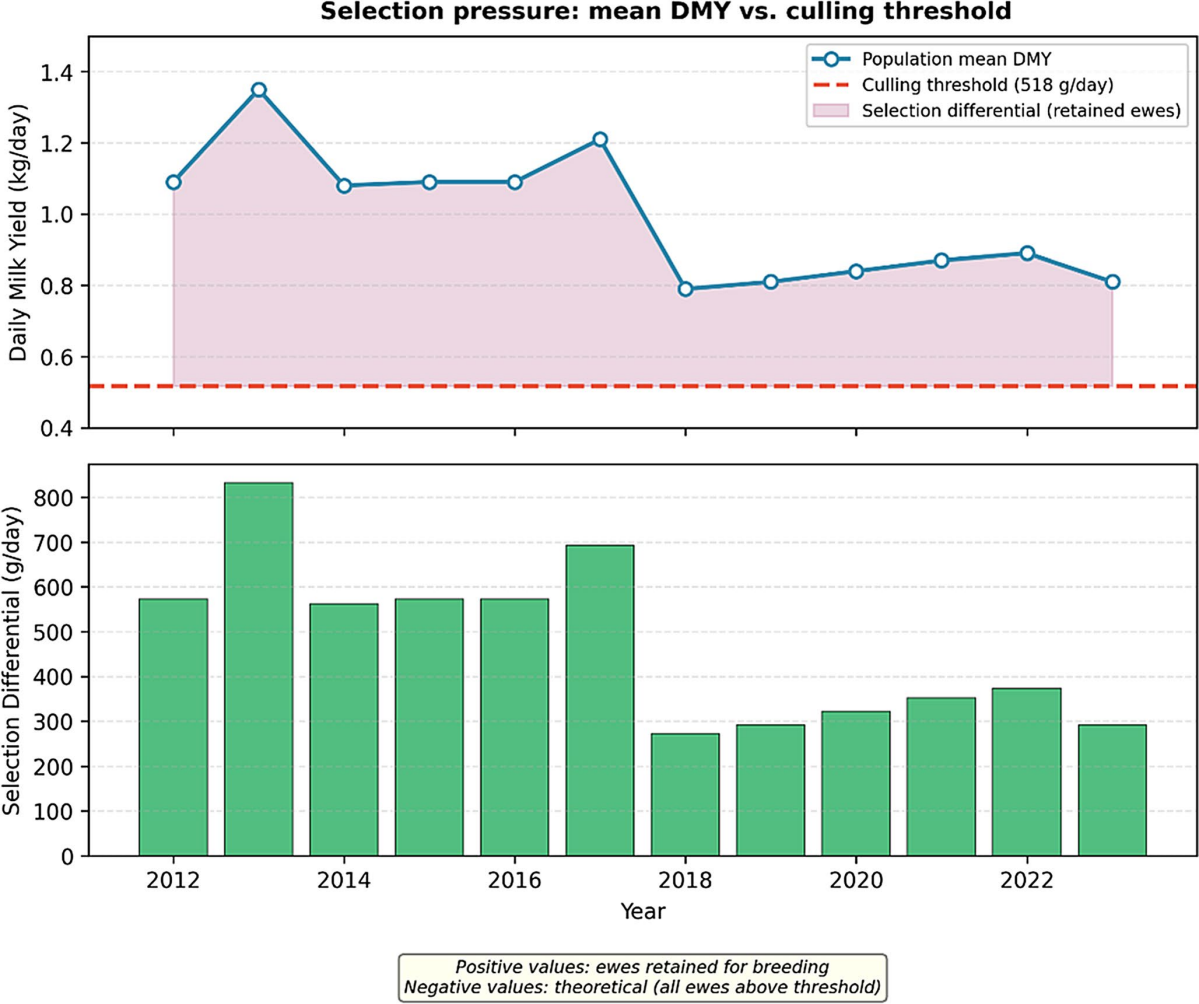
Illustration of selection pressure in the GSF Akkaraman population (2012–2023). The solid line represents annual mean daily milk yield (DMY, g day⁻¹); the dashed horizontal line indicates the culling threshold (517.5 g day⁻¹). The vertical distance between lines approximates the selection differential for retained ewes

The study was conducted in a nucleus breeding flock of 6,822 purebred Akkaraman ewes at Gözlü State Farm in Konya, Türkiye (38° 29’ N − 32° 27’ E), between 2012 and 2023. Selection intensity was maintained at approximately 20%, where the top 20% of ewes based on previous lactation records were retained for breeding. Culling criteria included daily milk yields below 517.5 g (500 ml) or failure to lamb annually (Fig. 3). Replacement ewes aged 2–6 years were used, and rams were selected based on twin birth and high maternal milk yield.

All sheep were provided with unrestricted access to potable water and natural mineral block licks. Ten days post-lambing, the ewes were permitted daily access to the pasture, except during the winter months from October to March. They were fed concentrated feed (300 g/ewe/day) and roughage (straw, etc.) throughout the winter. The ewes received approximately 700 g of wheat per ewe per day during the final third of gestation and throughout the lactation period. The sheep were feeding on wheat straw from July to September. Flush feeding commenced 15–20 days prior to the mating period in September and persisted throughout the initial 30 days of the mating period. The lambing period commenced in February (Güngör and Ünal 2020).

**Fig. 4:**
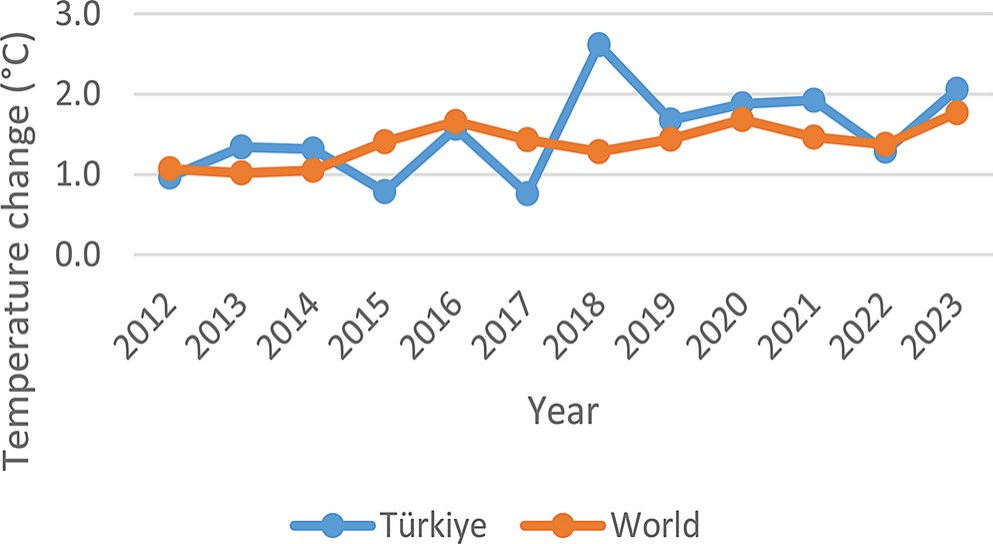
Temperature changes on lands (FAOSTAT 2025)

General feeding and management practices remained largely consistent throughout the study period. An exception occurred in 2013, when a temporary supplemental feeding strategy was implemented in response to an extreme drought event associated with increased air temperatures (0.8–2.6 °C) in terrestrial areas (Fig. 4). This additional supplementation was applied only during that year and was not repeated in subsequent years, even under comparable or more severe drought conditions. Therefore, no systematic or progressive changes in feeding management were introduced that could confound long-term trends in milk yield.

### Traits measured

Lactation Milk Yield (LMY) was standardized to 120 days to account for varying lactation lengths. Test-day yields were recorded on days 1, 45, 60, 75, 90, 105, and 120 of lactation. Milk composition and body weight were managed but not statistically analyzed in this report. Milk yield of ewes was measured in milliliters using measuring cups integrated into the milking system. All measurements were converted to grams using a density coefficient of 1.035 g/cm³ (Yardımcı and Özbeyaz 2001; Ünal et al. 2002; Koca et al. 2023). All animal handling and milking procedures were conducted in accordance with the ethical guidelines of the Gözlü State Farm and national animal welfare regulations.

### Statistical analyses

Statistical analyses were performed using IBM SPSS Statistics version 30.0 (IBM Corp. 2021). As this study evaluates the phenotypic outcomes of a practical breeding program, formal genetic parameter estimation (e.g., REML, BLUP) was not applied. Instead, descriptive statistics (mean, SD, SE) were calculated for annual yields. Quadratic spline interpolation (Python SciPy) was used to reconstruct lactation curves from test-day means. Consequently, heritability and breeding values were not estimated, and results represent phenotypic trends associated with the selection program.

Lactation milk yield was standardized to 120 days to allow consistent comparison across years and to minimize bias arising from variation in lactation length under extensive and semi-arid production conditions. In Akkaraman sheep and other indigenous fat-tailed breeds, the biologically active milking period is typically concentrated within the first 3–4 months of lactation (Akçapınar and Özbeyaz 2021), after which milk yield declines sharply due to nutritional limitations and management practices. Standardization to a 120-day lactation period therefore captures the physiologically relevant phase of milk production while reducing the influence of late-lactation variability. This approach is commonly applied in studies involving local sheep breeds managed under extensive systems. The mean milk yields from test days, obtained from annual SPSS analyses over twelve years, were the data source for the quadratic spline interpolation executed in Python. All lactation curves presented in this study were derived from quadratic spline interpolation applied to average milk yield on test days and not from individual animal records. This article exclusively employed the quadratic spline model due to the linear and cubic models yielding inconsistent lactation durations and negative milk yield.

A quadratic spline interpolation was conducted to estimate milk output across a 120-day lactation period. The research was performed using the Python programming language and the SciPy module. The analysis used milk yield measurements recorded on test days over a period of twelve years. The measurements were collected on days 1, 45, 60, 75, 90, 105, and 120 of lactation. No missing test-day records were present at the test-day mean level used for spline interpolation. On some test days, a small proportion of ewes could not be milked due to management-related reasons; however, milk yield calculations were based on test-day mean values derived from the available records. The proportion of unmilkable ewes did not exceed 5% of the total flock size in any given year; therefore, no data imputation or correction procedure was applied. Quadratic spline interpolation was used to estimate how much milk each day throughout a 120-day lactation period. The interp1d function from the SciPy package was used for this purpose. The interpolation approach allows us the ability to create a continuous function between certain data points and guess what the missing values are. We calculated the interpolated anticipated milk production estimates for each day from day 1 to day 120. Goodness-of-fit metrics were calculated to quantify the agreement between observed daily mean milk yields and values obtained from quadratic spline interpolation. Because the spline function was fitted deterministically to pass through all test-day mean points, the interpolation reproduced the observed values exactly. Consequently, the coefficient of determination (R²) equaled 1.00, and both the root mean square error (RMSE) and mean absolute error (MAE) were equal to zero. These metrics reflect the deterministic nature of spline interpolation rather than predictive model performance, and the primary objective of the approach was curve reconstruction and standardization of lactation yield rather than prediction. The visualization technique used the Matplotlib library.

## Results

Quadratic spline interpolation was used to forecast how much milk Akkaraman sheep will produce over 120 days. Annual lactation milk yield (LMY) and daily milk yield (DMY) values are presented as mean ± standard deviation (SD), with standard errors (SE) calculated to reflect inter-annual variability across the 12-year study period (Table 1).

**Table 1:** 120-days lactation milk yields of Akkaraman Sheep

Years	*n*	LMY (kg)	DMY (kg)
2012	633	130.21	1.09
2013	356	162.06	1.35
2014	670	129.36	1.08
2015	1,338	130.30	1.09
2016	684	131.24	1.09
2017	236	145.05	1.21
2018	466	94.54	0.79
2019	493	97.69	0.81
2020	527	100.85	0.84
2021	488	104.00	0.87
2022	478	107.16	0.89
2023	453	97.32	0.81
**All years mean (2012–2023)**	**6**,**822**	**119.15 ± 21.87 (SE = 6.31)**	**0.99 ± 0.18 (SE = 0.05)**

LMY: lactation milk yield (kg); DMY: daily milk yield (kg day⁻¹). Values are presented as mean ± SD, with SE indicating interannual variability. These values reflect phenotypic variation across years; formal genetic parameter estimation (e.g., heritability, breeding values) was not performed in this study

### Phenotypic trend analysis

**Fig. 5:**
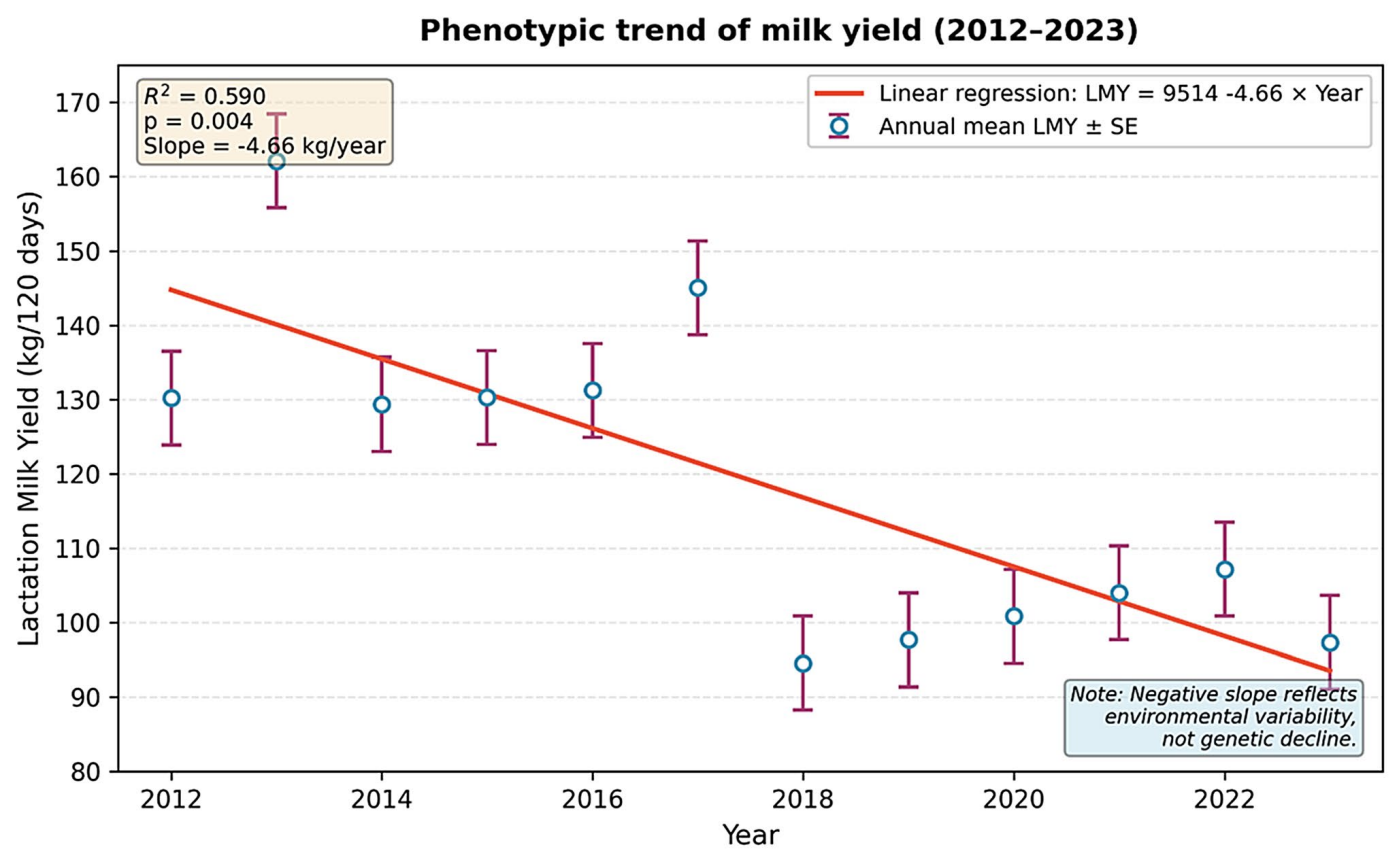
Phenotypic trend of annual mean lactation milk yield (LMY, kg/120 days) in the GSF Akkaraman population (2012–2023). The solid line represents the linear regression (LMY = 9514.067–4.657 × Year; R² = 0.590, *p* = 0.004). Error bars indicate standard error of annual means

To quantify the direction and magnitude of change in milk yield over the study period, a simple linear regression was performed with annual mean lactation milk yield (LMY) as the dependent variable and year (2012–2023) as the independent variable. The regression equation was: LMY (kg) = 9514.067–4.657 × Year with R² = 0.590 and *p* = 0.004 (Fig. 5). This indicates a statistically significant negative slope, reflecting an average phenotypic change of approximately − 4.66 kg per year. We acknowledge that this negative trend likely reflects interannual environmental variability (e.g., drought, feed availability/vegetation, temperature fluctuations; Fig. 4) rather than a decline in genetic potential. The long-term phenotypic yield pattern of the selection program is more accurately represented by comparing the earlier lactation means reports of 50–60 kg (Akçapınar and Özbeyaz 2021) and the late mean in this study (2023: 97.32 kg) within the context of stable management practices, as discussed in the Discussion section.

**Fig. 6:**
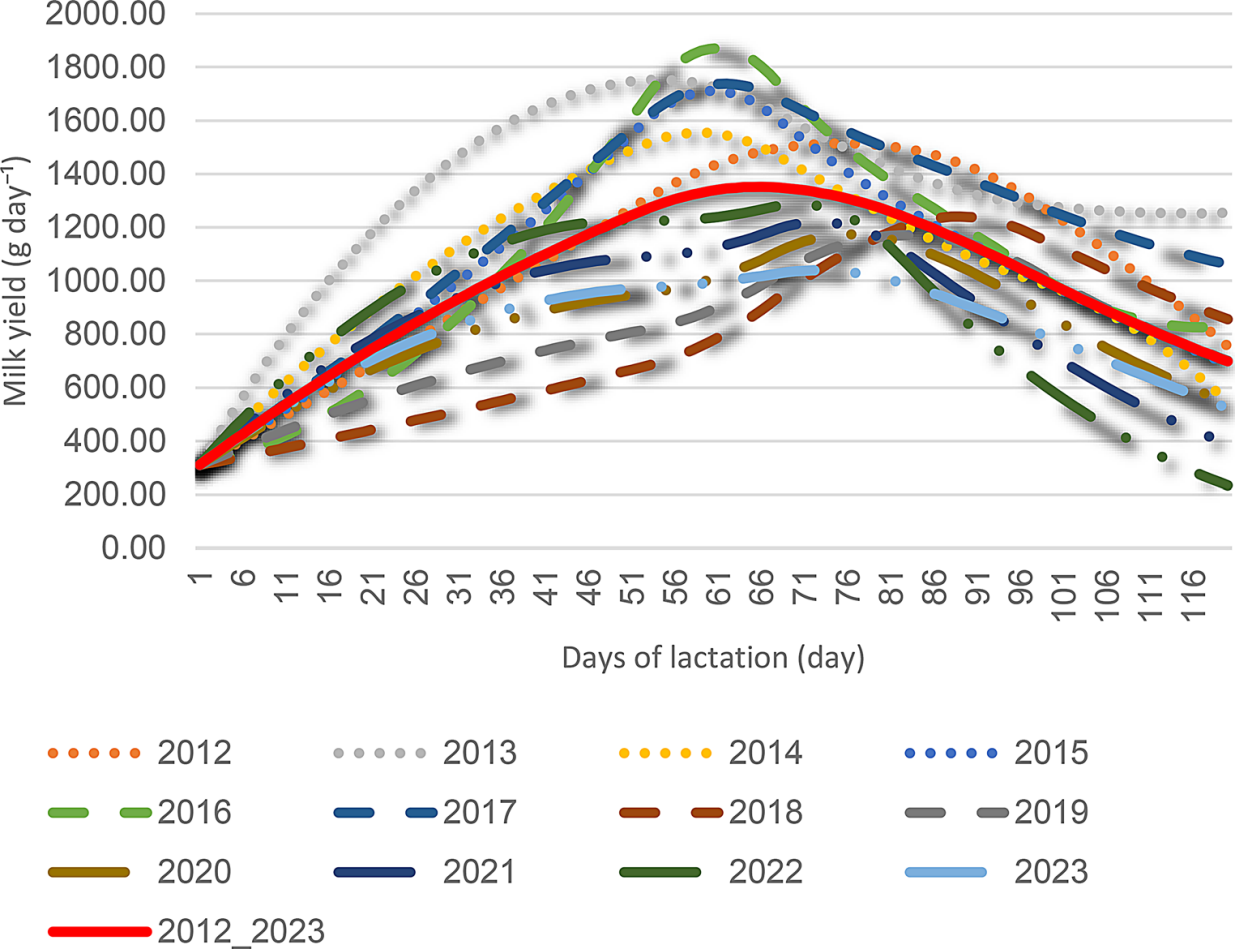
The curve was generated using test-day mean milk yield values (2012–2023)

**Fig. 7:**
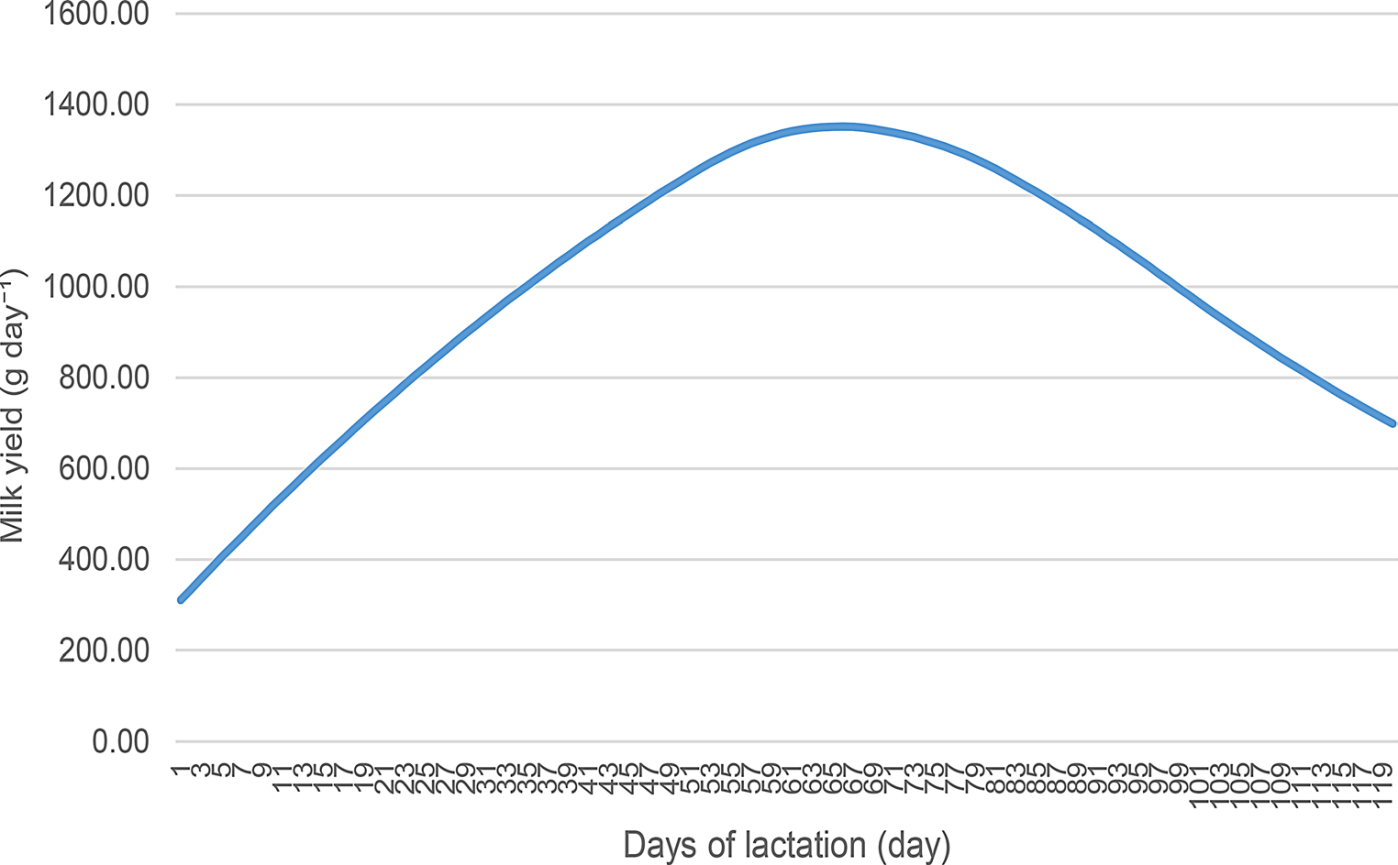
Akkaraman sheep 120-day lactation graphic

Figures 6 and 7 illustrate the annual and cumulative lactation graphs for Akkaraman sheep. The quadratic spline interpolation reproduced all observed test-day mean milk yield values exactly, resulting in a coefficient of determination (R²) of 1.00, with root mean square error (RMSE) and mean absolute error (MAE) values equal to zero.

## Discussion

The present study demonstrates that sustained phenotypic selection within the indigenous Akkaraman breed can lead to substantial improvement in milk yield without compromising adaptation to semi-arid production conditions. The long-term and directional nature of the observed improvement suggests that methodical within-breed selection can effectively enhance dairy performance in local fat-tailed sheep populations.

Milk yield performance in this study was evaluated using long-term phenotypic records without the application of formal genetic–environmental partitioning models. Management and feeding practices were largely consistent throughout the study period, with the exception of a short-term supplementation event implemented in response to an extreme drought in 2013. Although interannual climatic variability may have contributed to short-term fluctuations in milk yield, these effects were not consistently associated with sustained increases in production. In contrast, the cumulative improvement observed across multiple generations under a structured selection scheme suggests an important contribution of genetic gain. Nevertheless, the absence of mixed-model or breeding value–based analyses limit the ability to quantitatively separate genetic and environmental effects, and the results should be interpreted accordingly. The phenotypic regression analysis indicated a statistically significant negative slope (–4.66 kg/year, *p* = 0.004); however, this trajectory likely reflects short-term environmental fluctuations (e.g., drought events, feed variability) rather than the program’s long-term directional selection outcome, which is better captured by comparing period means under consistent management. An additional limitation of the present study is the use of average milk yield values from test days combined with spline interpolation to reconstruct lactation curves. While this approach is suitable for standardization and comparison across years, it does not capture individual-level variability in lactation dynamics and may smooth short-term fluctuations associated with management or environmental stressors. Therefore, the reconstructed curves should be interpreted as population-level representations rather than precise individual lactation profiles. The observed trends should therefore be considered descriptive phenotypic patterns rather than formally estimated genetic trends. Despite this limitation, the consistency and persistence of improvement under relatively stable management conditions are compatible with the expected outcomes of long-term phenotypic selection. Although genetic parameters were not estimated, the sustained improvement over multiple generations under consistent management aligns with expected responses to directional phenotypic selection (Falconer and Mackay 1996).

### Mechanism of improvement

It is critical to distinguish between the sources of observed performance changes. While the selection program applied consistent phenotypic pressure for milk yield, the negative regression slope observed in the Results section (–4.66 kg/year) indicates that year-to-year fluctuations were predominantly driven by environmental variability (e.g., drought events, feed availability) rather than linear genetic gain. Therefore, the reported “improvement” in this study is primarily contextualized against historical breed averages (e.g., 50–60 kg in earlier reports vs. 119 kg in this study) rather than a within-study temporal trend. Without pedigree-based BLUP analyses, we cannot quantify the additive genetic component; however, the sustained higher performance relative to historical baselines suggests that phenotypic selection, combined with stable management, can unlock latent production potential in indigenous breeds.

### Comparison with other breeds

Although milk yields achieved in the selected Akkaraman population remain lower than those of highly specialized international dairy breeds (e.g., Lacaune, Assaf), they are competitive within dual-purpose and pasture-based production systems typical of Mediterranean and semi-arid regions (Table 2). Specifically, the GSF Akkaraman population’s mean daily milk yield (~ 993 g/day) exceeds reported values for native Awassi and Bafra breeds under similar extensive conditions (Table 2). This benchmarking highlights that within-breed selection can bridge the productivity gap between indigenous fat-tailed breeds and specialized dairy populations without compromising adaptation traits often diminished in high-input systems. Importantly, this improvement was achieved without crossbreeding, preserving key breed characteristics such as robustness, maternal ability, and tolerance to extensive management—traits often diminished in high-input dairy systems. This finding aligns with current approaches to sustainable intensification, which emphasize the utilization of resilient local breeds capable of adapting to climate variability while maintaining acceptable productivity.

The shape of the lactation curve further indicates a shift toward a more persistent, dairy-type lactational pattern compared with the rapid post-peak decline commonly observed in meat-oriented fat-tailed breeds (Table 3). Improved lactation persistency enhances economic stability in smallholder dairy systems by extending the effective milking period and reducing seasonal constraints. From a climate-smart production perspective, higher milk yield per ewe allows a greater proportion of maintenance energy to be allocated to milk synthesis, thereby reducing methane emissions per unit of milk produced. Even in the absence of direct methane measurements, this dilution effect—where fixed maintenance-related emissions are distributed over higher milk output—is well recognized in ruminant production systems. Consequently, the observed increase in milk yield per ewe implies improved emission efficiency and supports climate-smart livestock production strategies under semi-arid conditions.

### Sustainability and trade-offs

While increased milk yield is desirable, intensifying production in dual-purpose breeds may incur antagonistic effects on other functional traits. High-yielding selection programs in livestock have been associated with potential trade-offs, including reduced fertility (David et al. 2008; Sundrum 2015), shortened productive lifespan, and increased susceptibility to metabolic disorders (Sundrum 2015). Although the GSF Akkaraman population maintained adaptation to semi-arid conditions during this study, long-term sustainability requires monitoring these antagonistic correlations. Future breeding goals should therefore balance milk yield with fertility, longevity, and disease resistance to prevent erosion of resilience traits that are critical for low-input systems.

**Table 2:** Mean Daily Milk Yield (DMY) of Sheep Breeds Grouped by Origin (g day⁻¹)

**Akkaraman, Crossbreeds and Varieties (ACV)**		
**Reference**	**Breed**	**DMY (g day⁻¹)**
Karadavut 2020	Akkaraman	441.23
Duman et al. 2022	Akkaraman	619.70
Kahraman and Özkul 2020	Akkaraman	683.61
Güngör et al. 2022	Akkaraman	700.77
**This study 2025**	**Akkaraman GSF**	**992.90**
Kahraman and Özkul 2020	BafraXAkkaraman	753.17
Güngör et al. 2022	BAKB1	773.40
Koyun et al. 2021	Karakaş	656.03
Yağcı and Baş 2024	Şavak	616.50
Bayrıl et al. 2023	Zom	676.40
**Other Native Sheep Breeds (ONSB)**		
**Reference**	**Breed**	**DMY (g day⁻¹)**
Koyun et al. 2021	Norduz	667.08
Kahraman and Özkul 2020	Bafra	849.76
Şeker et al. 2024	Bafra	868.60
Koncagül et al. 2024	Turkish Awassi	494.00
Şeker et al. 2022	Turkish Awassi	659.49
Kahraman et al. 2022	Turkish Awassi	915.33
Shihab 2024	Turkish Awassi	981.00
Akgün and Koyuncu 2020	Kıvırcık	495.50
Vanlı and Kaygısız 2024	Morkaraman	560.00
**Other Sheep Breeds (OSB)**		
**Reference**	**Breed**	**DMY (g day⁻¹)**
Gebreslase et al. 2024	50%AwassiXMenz	1020.00
Gebreslase et al. 2024	75%AwasiXMenz	1060.00
Toral et al. 2023	Assaf	2600.00
Li et al. 2022	Assaf	2900.00
Al-Juwari et al. 2024	Awassi	570.80
Ayadi et al. 2024	Awassi	630.00
Awawdeh 2024	Awassi	719.75
Rashid et al. 2024	Awassi	992.76
Obeidat et al. 2024	Awassi	1435.00
Gebreslase et al. 2024	Awassi	1690.00
Li et al. 2022	Awassi	3710.00
Pugliano et al. 2024	Bagnolese	717.46
Abadi and Heydari 2024	Baluchi*	856.25
Rashid et al. 2024	Barki	583.57
Stocco et al. 2024	Comisana	840.00
Brandão et al. 2024	Dorper	436.82
Ma et al. 2024	Hu	1738.80
Makovicky et al. 2024b	ImprovedValachian(IV)	378.89
Makovicky et al. 2024a	ImprovedValachian(IV)	525.26
Makovicky et al. 2024b	IVXEF(25%)	363.08
Makovicky et al. 2024b	IVXEF(50%)	439.54
Makovicky et al. 2024a	IVXLacune50%(LC)	601.75
Makovicky et al. 2024a	IVXLC75%	579.91
Karageorgou et al. 2024	Karagouniki	810.41
Ibrayev et al. 2022	Kazakh Fat Tailed	770.00
Rashid et al. 2024	Kermanian	305.93
Antunović et al. 2024	Lacune	1399.08
Bernard et al. 2024	Lacune	1987.20
Li et al. 2022	Lacune	2500.00
Li et al. 2022	Laxta	3030.00
Gebreslase et al. 2024	Menz	810.00
Ayadi et al. 2024	Najdi	750.00
Matar et al. 2024	Najdi	1570.00
Marshall et al. 2024b	NZDS	589.95
Marshall et al. 2025a	NZDS	683.10
Marshall et al. 2025b	NZDS	589.95
Li et al. 2022	OstFriz	2290.00
Rashid et al. 2024	PelibueyXKatahdin	738.50
Petrović et al. 2024	Pirot	529.84
Mazareei et al. 2024	Qezel	506.25
Crosby-Galvan et al. 2024	Ramboilet	1020.00
Brandão et al. 2024	Santa İnes	613.75
Carta et al. 2023	Sarda	950.00
Li et al. 2022	Sarda	2500.00
Smeti et al. 2024	Sarda	574.94
Makovicky et al. 2024a	Tsigai	328.20
Makovicky et al. 2024a	TXLC	499.08
Di Miceli et al. 2024	ValleDelBelice	1243.50

ACV: Akkaraman, Crossbreeds and Varieties; ONSB: Other Native Sheep Breeds; OSB: Other Sheep Breeds; DMY: Daily milk yield; GSF: Gözlü State Farm.* Fat-tailed breed phenotypically similar to Akkaraman.

**Table 3:** Mean Lactation Milk Yield (LMY) of Sheep Breeds Grouped by Origin (kg)

**Akkaraman, Crossbreeds and Varieties (ACV)**		
**Reference**	**Breed**	**LMY (kg)**
Karadavut 2020	Akkaraman	47.63
Duman et al. 2022	Akkaraman	55.00
Karaman and Özkul 2020	Akkaraman	99.57
Güngör et al. 2022	Akkaraman	109.46
**This study 2025**	**Akkaraman GSF**	**119.15**
Aşkan and Aygün 2020	Akkaraman Crossbreed	99.78
Kahraman and Özkul 2020	BafraXAkkaraman GSF	112.52
Güngör et al. 2022	BAKB1	124.55
Yağcı and Baş 2024	Şavak	88.80
Bayrıl et al. 2023	Zom	123.45
**Other Native Sheep Breeds (ONSB)**		
**Reference**	**Breed**	**LMY (kg)**
Hasan and Ceyhan 2024	Turkish Awassi Mutation	147.44
Hasan and Ceyhan 2024	Turkish Awassi	123.99
Kahraman and Özkul 2020	Bafra	126.40
Şeker et al. 2024	Bafra	156.86
Koncagül et al. 2024	Turkish Awassi	88.89
Gül and Oflaz 2021	Turkish Awassi	125.90
Şeker et al. 2022	Turkish Awassi	138.59
Erol et al. 2020	Karakul	104.85
Akgün and Koyuncu 2020	Kıvırcık	104.40
Vanlı and Kaygısız 2024	Merinos	65.50
Vanlı and Kaygısız 2024	Morkaraman	79.35
Vanlı and Kaygısız 2024	MorkaramanXMerinos	74.70
Koca et al. 2023	Norduz	137.20
Tüney Bebek and Keskin 2021	Southkaraman	37.70
**Other Sheep Breeds (OSB)**		
**Reference**	**Breed**	**LMY (kg)**
Salman et al. 2024	AfecAwassi	185.52
Salman et al. 2024	Assaf	215.20
Li et al. 2022	Assaf	506.00
Salman et al. 2024	Awassi	167.83
Li et al. 2022	Awassi	460.00
Salman et al. 2024	AwassiXAssaf	222.22
Stankov and Karakolev 2024	Awasssi	123.17
Pugliano et al. 2025	Bagnolese	107.00
Ibrahimov and Maharramov 2024	Balbas*	100.00
Stancheva et al. 2024	BDSP	119.29
Salman et al. 2024	ImprovedAwassi	171.35
Evodullaevna and Rahimovich 2024	Jaydari	41.30
Evodullaevna and Rahimovich 2024	JaydariXHisar	44.90
Sodi et al. 2024	Lacune	220.00
Li et al. 2022	Lacune	454.00
Li et al. 2022	Laxta	446.00
Sodi et al. 2024	Messese	108.00
Marshall et al. 2024a	NZDS	86.10
Li et al. 2022	OstFriz	700.00
Sodi et al. 2024	Sarda	180.00
Li et al. 2022	Sarda	376.00

ACV: Akkaraman, Crossbreeds and Varieties; ONSB: Other Native Sheep Breeds; OSB: Other Sheep Breeds; LMY: Lactation milk yield; GSF: Gözlü State Farm

* Fat-tailed breed phenotypically similar to Akkaraman

## Conclusion

This 12-year selection study demonstrates that sustained within-breed phenotypic selection can substantially improve milk yield in the indigenous Akkaraman fat-tailed sheep without compromising its adaptation to semi-arid production systems. The selected GSF Akkaraman population achieved standardized daily and lactation milk yields that are comparable to, and in some cases exceed, those reported for other native and crossbred dairy sheep populations in Türkiye. These results indicate that the Akkaraman breed can function effectively as a dairy-type ecotype under low-input conditions.

Based on the improved performance relative to historical breed averages, the selected GSF Akkaraman population represents a viable alternative to crossbreeding strategies that rely on imported germplasm. The combination of enhanced milk production, maintained environmental resilience, and compatibility with extensive management supports its suitability for sustainable dairy sheep production in semi-arid regions.

Although pedigree records were maintained throughout the study, formal inbreeding coefficient calculations were beyond the scope of this phenotypic evaluation; therefore, future genomic monitoring is essential to ensure long-term genetic diversity.

Accordingly, the following recommendations are proposed: (i) evaluation for formal recognition of the GSF Akkaraman population as a distinct dairy ecotype pending genetic validation; (ii) integration of genomic tools to accelerate genetic gain while monitoring inbreeding coefficients and controlling genetic diversity; (iii) expansion of the breeding nucleus to additional state farms to increase genetic diversity and dissemination; and (iv) implementation of life-cycle assessment studies to quantitatively evaluate environmental and economic performance relative to specialized imported dairy breeds.

Overall, the results provide empirical support for a regionally adapted, climate-smart approach to improving sheep milk production based on the genetic enhancement of local breeds, offering a promising pathway for Türkiye and similar agro-ecological zones pending further genetic characterization.

## Data Availability

The supplementary data can be available from the corresponding author upon a reasonable request.
